# Al-Tolerant Barley Mutant *hvatr.g* Shows the ATR-Regulated DNA Damage Response to Maleic Acid Hydrazide

**DOI:** 10.3390/ijms21228500

**Published:** 2020-11-12

**Authors:** Joanna Jaskowiak, Jolanta Kwasniewska, Miriam Szurman-Zubrzycka, Magdalena Rojek-Jelonek, Paul B. Larsen, Iwona Szarejko

**Affiliations:** 1 Plant Cytogenetics and Molecular Biology Group, Institute of Biology, Biotechnology and Environmental Protection, Faculty of Natural Sciences, University of Silesia in Katowice, Jagiellonska 28, 40-032 Katowice, Poland; joanna_jaskowiak@wp.eu (J.J.); magdalena.rojek@us.edu.pl (M.R.-J.); 2 Plant Genetics and Functional Genomics Group, Institute of Biology, Biotechnology and Environmental Protection, Faculty of Natural Sciences, University of Silesia in Katowice, Jagiellonska 28, 40-032 Katowice, Poland; miriam.szurman@us.edu.pl (M.S.-Z.); iwona.szarejko@us.edu.pl (I.S.); 3 Department of Biochemistry, University of California, Riverside, CA 92521, USA; paull@ucr.edu

**Keywords:** aluminum toxicity, ATR, barley, cell cycle, DDR pathway, DNA damage, maleic acid hydrazide

## Abstract

ATR, a DNA damage signaling kinase, is required for cell cycle checkpoint regulation and detecting DNA damage caused by genotoxic factors including Al^3+^ ions. We analyzed the function of the *HvATR* gene in response to chemical clastogen-maleic acid hydrazide (MH). For this purpose, the Al-tolerant barley TILLING mutant *hvatr.g* was used. We described the effects of MH on the nuclear genome of *hvatr.g* mutant and its WT parent cv. “Sebastian”, showing that the genotoxic effect measured by TUNEL test and frequency of cells with micronuclei was much stronger in *hvatr.g* than in WT. MH caused a significant decrease in the mitotic activity of root cells in both genotypes, however this effect was significantly stronger in “Sebastian”. The impact of MH on the roots cell cycle, analyzed using flow cytometry, showed no differences between the mutant and WT.

## 1. Introduction

Genome stability is essential for the proper maintenance and transmission of genetic information. Plants are constantly exposed to stress conditions that can damage their DNA [1]. A highly conserved DNA damage response (DDR) pathway involves activation of the cell cycle checkpoints and specific DNA repair factors in response to stress conditions [2]. The main activators of DDR are two phosphoinositide 3-kinase-like kinases: ATM (Ataxia Telangiectasia Mutated) and ATR (ATM and Rad3-related). The ATM-related pathway is known to be activated upon DNA double strand breaks (DSBs), whereas the ATR-related pathway upon single stranded DNA recognition [3]. Plant stem cells are particularly sensitive to DNA damage in comparison to other plant tissue types and thus specific mechanisms are established to protect them. 

Al^3+^ toxicity is a severe agricultural problem, as it limits crop productivity in acidic soils (pH 5.5 and lower) by the inhibition of plant root growth [4,5,6]. The mechanism of Al toxicity is still not fully elucidated, and the complexity of the multilevel molecular mechanisms is indicated in many studies [7,8]. Among the potential targets for Al toxicity are apoplastic and symplastic sites [9,10]. One of the most important targets for Al^3+^ is DNA, by having effects on gene expression and chromosome structure. 

The role of the plant DNA Damage Response (DDR) in reaction to metal toxicity is well documented [2]. It has been shown in Arabidopsis that the DDR pathway is also involved in response to Al [11]. A study on the mechanisms responsible for the Al-dependent root growth inhibition indicated that Ataxia Telangiectasia and Rad-3-related (ATR), a DNA damage signaling kinase, is required for monitoring DNA integrity following Al exposure [12]. ATR is activated when persistent ssDNA is accumulated in the nucleus [1] and is responsible for the cell cycle checkpoint regulation and detection of DNA damage that leads to stalled replication forks [13]. ATR responds to a large variety of genotoxic stresses that all slow down DNA polymerases, leading to accumulation of single stranded DNA [14]. In this context, among the metals, Al is one of the best studied, because it is very abundant in the Earth’s crust and is one of the primary growth limiting factors in agriculture. Al was shown to act as a mild genotoxic agent that induced DNA damage in Arabidopsis [8,15] and barley [16], as well as root growth inhibition with the involvement of the ATR-related DDR pathway [12,17,18]. The Arabidopsis *atr-1, atr-2* and *atr-3* knockout mutants were sensitive to various replication-blocking and genotoxic agents, such as hydroxyurea (HU), aphidicolin and UV-B light, due to the failure to initiate DNA repair [19]. However, the loss of the ATR function leads to increased tolerance to micromolar concentrations of Al that reflect the doses typically found in natural conditions in acidic soils. The Al-tolerant phenotype of *atr* mutants was due to the failure to arrest the cell cycle progression [12]. 

In our previous studies, we developed a barley mutant *hvatr.g* carrying a G6054A missense mutation in the *HvATR* gene [20]. The mutation led to the substitution of a highly conserved amino acid in the UME domain of ATR protein (G1015S). Although the UME domain (named after three kinases in which it was found: UVSP PI-3, MEI-41 and ESR-1) [21] is of unknown function, it appears to be involved in Al response in Arabidopsis [12]. The barley *hvatr.g* mutant was identified in the *Hor*TILLUS population developed at the University of Silesia in Katowice [22]. In control conditions it showed a high level of accumulated DNA damage whose frequency significantly increased after exposure to Al treatment. Furthermore, no cell cycle arrest was observed in the *hvatr.g* mutant after Al treatment, which resulted in the Al tolerance manifested by the lack of root growth inhibition. That study clearly indicated that the *hvatr.g* mutant has an impaired mechanism of DNA damage response [20].

The aim of the presented study was to analyze the possible role of the ATR-dependent DDR pathway in response to other DNA-damaging agents and thus to verify whether this mechanism is universal or is Al-specific only. In this study we provide the comprehensive characterization of the response of the *hvatr.g* mutant to DNA damage caused by the chemical agent maleic acid hydrazide (MH). MH, chemically defined as 1,2-dihydro-3,6-pyridazinedione, is a clastogenic and mutagenic agent that may cause spindle fiber defects [23,24]. MH is an effective chromosome-breaking agent in higher plants and it is commonly used as a reference mutagen in plant mutagenesis aimed to induce DNA breakage and chromosome aberrations. MH is proved to have DNA-damaging effects, as shown by the comet assay [25,26] and clastogenic effects in barley [24,27,28].

There is no molecular evidence that MH acts directly on DNA, but its effect on S phase proteins has been confirmed by inhibition of the synthesis of nucleic acids and the enzymes that are involved in the formation of the mitotic spindle [29,30,31]. It was proved that MH affects the S phase in barley [32]. 

## 2. Results

### 2.1. Response of hvatr.g Mutant and Its Parental Line to MH Treatment 

#### 2.1.1. Mitotic Activity 

**Figure 1:**
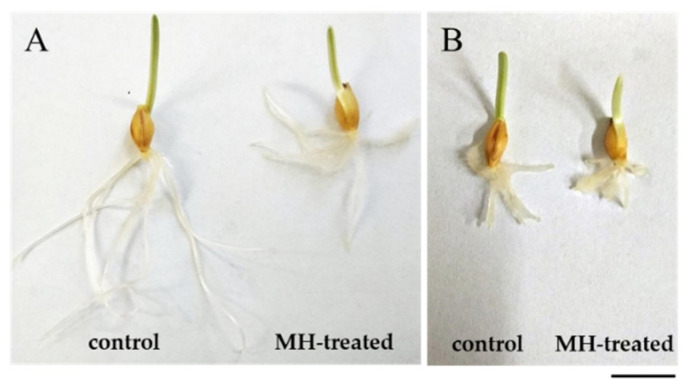
The representative 3-day-old seedlings of barley: cv. “Sebastian” (**A**) and *hvatr.g* mutant (**B**): control and treated with 4 mM MH. Bar 1 cm.

The differences in the length of “Sebastian” and *hvatr.g* mutant roots were observed both in control conditions and after MH treatment. In control conditions, the mean length of the roots of 3-day-old “Sebastian” seedlings was 4.1 cm ± 0.5, while the length of mutant seedlings was 1.2 cm ± 0.2. A significant decrease of root length was observed after treatment with 4 mM MH of both genotypes (Figure 1), however the mean length of “Sebastian” seminal roots decreased by almost 50%, while seminal roots of the mutant decreased by only 25%.

**Figure 2:**
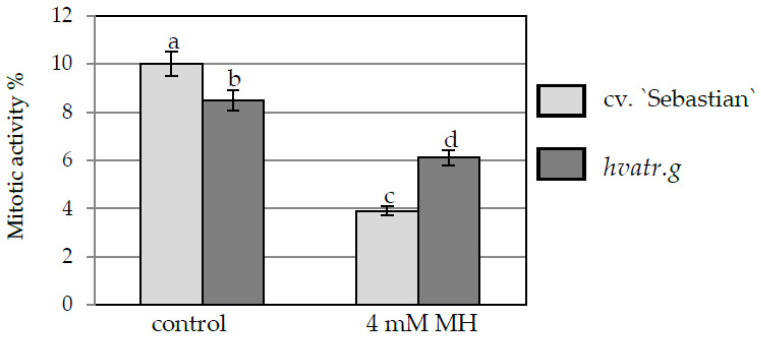
Mitotic activity of root cells of barley seedlings of the *hvatr.g* mutant and cv. “Sebastian”, control and MH-treated. Error bars represent the standard deviations of the mean. Statistical analyses were performed using ANOVA (*p* < 0.05) followed by Tukey’s honestly significant difference test (Tukey HSD test, *p* < 0.05). Statistically significant differences are indicated by different letters.

The reduction of root length in cv. “Sebastian” and *hvatr.g* mutant was in agreement with decreased mitotic activity observed in the root meristems of both genotypes. Treatment with 4 mM MH reduced the mitotic activity of both lines (Figure 2), however the effect was different between “Sebastian” and the *hvatr.g* mutant. The mitotic activity of “Sebastian” root meristematic cells in control conditions was 10%, whereas after MH treatment it decreased to 3.9% (2.6-fold difference). Although much lower than for “Sebastian”, a significant decrease in the mitotic activity from 8.5% to 6.1% (1.4-fold difference) was also seen for *hvatr.g* mutants roots. It should be noted that even though *hvatr.g* mutant showed a lower mitotic activity and shorter roots than WT “Sebastian” already under control conditions, its mitotic activity was significantly higher than “Sebastian” in the presence of MH (Figure 1 and Figure 2).

#### 2.1.2. DNA Damage

##### Frequency of Cells with Micronuclei

**Figure 3:**
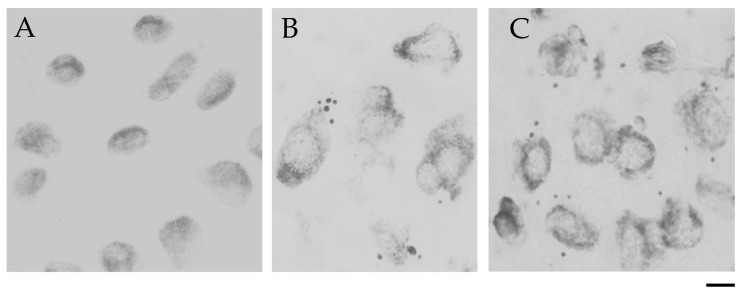
Cytological effects of MH in root cells of barley seedlings of the cv. “Sebastian” and *hvatr.g* mutant. (**A**) Control nuclei without micronuclei; (**B**,**C**) Nuclei with micronuclei after MH treatment: (**B**) cv. “Sebastian” (**C**) *hvatr.g* mutant. Bar 20 µm.

One of the cytogenetic effects of MH treatment in the meristematic root cells was the formation of micronuclei (Figure 3), which were observed in cv. “Sebastian” (Figure 3B), as well as in *hvatr.g* mutant (Figure 3C). 

**Figure 4:**
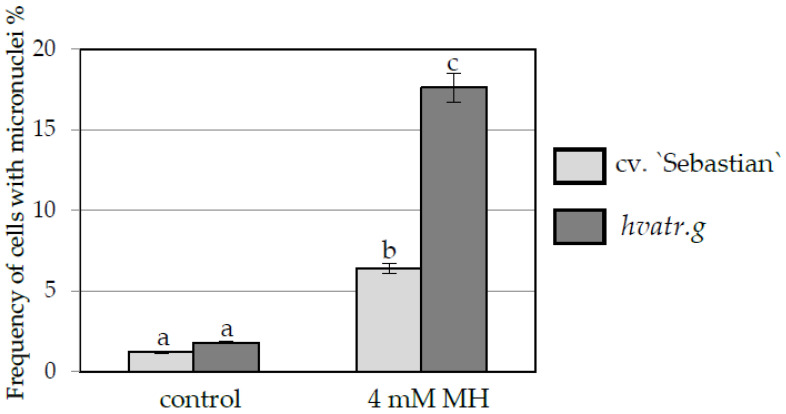
Frequency of cells with micronuclei in root of barley seedlings of the *hvatr.g* mutant and cv. “Sebastian”, control and MH-treated. Error bars represent the standard deviations of the mean. Statistical analyses were performed using ANOVA (*p* < 0.05) followed by Tukey’s honestly significant difference test (Tukey HSD test, *p* < 0.05). Statistically significant differences are indicated by different letters.

**Figure 5:**
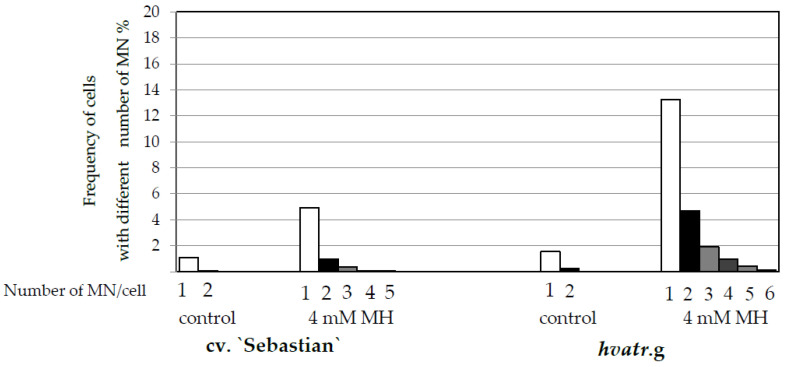
Frequencies of cells with different number of micronuclei (MN) in root cells of barley seedlings of the *hvatr.g* mutant and cv. “Sebastian”, control and MH-treated. The number of micronuclei is shown below the X axis.

Similar low levels of cells with micronuclei were observed in the control “Sebastian” and *hvatr.g* mutant roots (1.2% and 1.8% respectively). A significant increase in the frequency of cells with micronuclei was observed after 4 mM MH treatment, namely to 6.4% in “Sebastian” roots and to a much higher level, 17.6% in *hvatr.g* roots (Figure 4). In the cells that possess micronuclei, their number was evaluated. It ranged from 1 to 2 in control roots of “Sebastian” and *hvatr.g* mutant, while after MH treatment it increased up to 5 in “Sebastian” and 6 in *hvatr.g* roots (Figure 5). 

##### TUNEL Test

**Figure 6:**
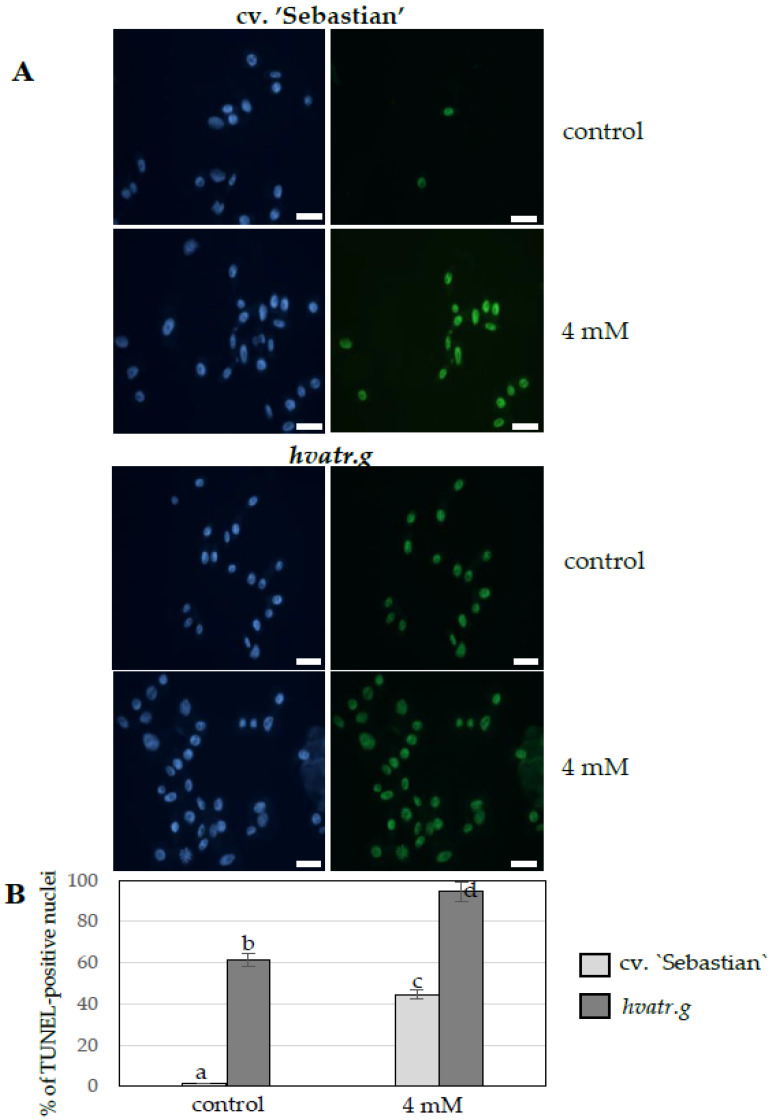
Results of TUNEL test in root cells of barley seedlings of the *hvatr.g* mutant and cv. “Sebastian”, control and MH-treated. (**A**) Examples of damaged nuclei observed in control “Sebastian” and *hvatr.g* roots and roots treated with 4 mM MH. Left images of DAPI-stained nuclei. Right images from the FITC channel. (**B**) Frequency of TUNEL-positive nuclei of root cells of barley seedlings of the *hvatr.g* mutant and cv. “Sebastian”, control and MH-treated. Statistical analyses were performed using ANOVA (*p* < 0.05) followed by Tukey’s honestly significant difference test (Tukey HSD test, *p* < 0.05). The significant differences (*p* < 0.05) between the groups are indicated by different letters.

To assess the rate of nuclei with DNA damage (DNA breaks), the TUNEL test was applied to control and MH-treated roots of “Sebastian” and the *hvatr.g* mutant. To determine the percentage of nuclei with DNA breaks, the root meristem cells were first stained with DAPI. The same nuclei with a green FITC fluorescence were considered to possess DNA damage (Figure 6A). In the control conditions, TUNEL-specific nuclei were observed in “Sebastian” root cells, with a low frequency of about 1.3%, while the *hvatr.g* mutant revealed as much as 61.3% of nuclei that showed TUNEL-specific fluorescence (Figure 6B). The control “Sebastian” roots that had been treated with DNase (positive control) showed TUNEL positive signals in 85% of the nuclei. The frequency of TUNEL-positive nuclei increased significantly after treatment with 4 mM MH in both genotypes and reached 44.4% in “Sebastian” and as much as 94.4% damaged nuclei in *hvatr.g* mutant. 

#### 2.1.3. Cell Cycle Analysis

**Figure 7:**
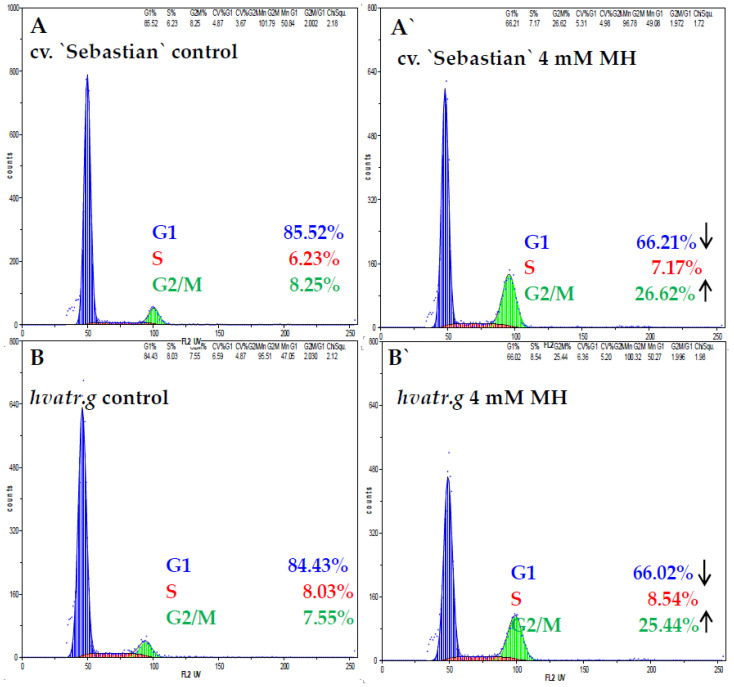
Flow cytometric analysis of the cell cycle in roots of barley cv. “Sebastian” (**A**,**A’**) and *hvatr.g* mutants (**B**,**B’**). (**A**,**B**) control roots, (**A’**,**B’**) 4 mM MH treated roots.

Flow cytometry analysis was used to evaluate the effect of MH on the cell cycle in meristematic root cells of both genotypes. Cell cycle profiles were similar in the control “Sebastian” and *hvatr.g* mutant roots (Figure 7). The frequency of the control root meristematic cells in the G1 phase was about 85% in both “Sebastian” and *hvatr.g* mutants. Only 6.2% of “Sebastian” cells and 8.0% of mutant cells were in the S phase and in the G2—7.5% and 8.2%, respectively. After treatment with MH the cell cycle profiles changed, although they did not differ from each other in the “Sebastian” and *hvatr.g* mutants. Root cells were still predominantly in G1, however the frequency of G1 cells decreased to about 66%. At the same time the frequency of cells in G2/M significantly increased in both genotypes. 

## 3. Discussion

In plants, ATR is a serine/threonine kinase that plays a role in activation of the DDR pathway by phosphorylating SOG1 (Suppressor of Gamma Radiation 1)—a central DDR transcription factor. The activation of this pathway in response to DNA damage leads to cell cycle arrest and activation of DNA repair processes [33]. Previously we described the *hvatr.g* mutant carrying a missense mutation in the *HvATR* gene and compared the cytogenetic response of the mutant and its parental line “Sebastian” to Al treatment [20]. We confirmed, using TUNEL and chromosomal aberration tests, that *hvatr.g* is impaired in the DDR pathway and accumulates DNA damage when grown in control hydroponics. In the presented studies, in which MH was used as a mutagen, the conditions of control plants were different from the previous experiment—seeds of the analyzed genotypes were pre-soaked for 8 h in H_2_O, then soaked for 3 more hours in H_2_O as a control to 3 h MH treatment, and the seedlings were grown in Petri dishes on a wet tissue paper for 72 h. Under these control conditions, less stressful than the growth in hydroponics, the *hvatr.g* mutant also showed a very high frequency (61.3%) of nuclei with DNA nicks and breaks compared to only 1.3% of TUNEL-positive nuclei observed in ”Sebastian” roots. These results are consistent with previous studies and clearly confirm that the *hvatr.g* mutant exhibits DNA damages even without any treatment with a genotoxic agent. However, in control conditions the DNA damages (particularly DSBs) have been efficiently repaired in the mutant, because the percentage of cells with micronuclei was at a similar low level in both genotypes (1.8% and 1.2% in the mutant and “Sebastian”, respectively). The TUNEL test shows DNA breaks and nicks, while the micronuclei index shows the DNA fragments that arose from the double strand breaks that had not been repaired and thus it indicates the efficient repair of DSBs in control conditions probably through the ATM-related DDR pathway.

In this study we described the *hvatr.g* mutant in the context of its response to the chemical clastogen maleic acid hydrazide (MH). MH affects the synthesis and processing of rRNA and may also act as an inhibitor of the synthesis of nucleic acids and enzymes that are involved in the mitotic spindle, which causes multipolar anaphases, lagging chromosomes and chromosome breaks [30]. MH acts only during S-phase [27,34]. However, the exact mechanism of its action is not fully understood and some of the effects caused by MH are not correlated with any particular cell cycle phase. It has not been proven that the mechanism of MH action has a direct effect on DNA [31], whereas Al^3+^, as a positive ion, has a potential to bind DNA directly to PO_4_^3-^ residues. Consequently, our analyses with the *hvatr.g* mutant describes the possible role of the ATR-dependent DDR pathway in response to other DNA-damaging agents and thus demonstrates the critical role of ATR in barley for maintenance of genome integrity. 

We demonstrated that MH treatment of the *hvatr.g* mutant increased the frequency of cells with DNA damage observed as micronuclei to 17.6%, compared to 6.4% in its parent variety. Moreover, the TUNEL test revealed an extremely high percentage of nuclei with DNA nicks and breaks (94%) in the mutant root meristem cells after MH treatment, compared to 44.4% of damaged nuclei in “Sebastian”. It is surprising that the very high frequency of TUNEL-positive nuclei in the mutant grown in control conditions (61%) was not accompanied by the high frequency of micronuclei (only 1.8%, which is a level not different from “Sebastian”), suggesting that DNA double strand breaks in the mutant are largely repaired, which is consistent with the notion that DSB sensing and repair mainly involves ATM, not ATR. However, after MH treatment, the extremely high frequency of TUNEL-positive nuclei in *atr.g* mutant correlated with the more frequent presence of micronuclei, which suggests that when challenged with higher rates of DNA damage such as following MH treatment, the mutant cannot repair the DNA damage effectively. Our results show that the ATR-related DDR pathway is required for the repair of MH-induced DNA damages in barley although in an unknown manner. Notwithstanding, the cell cycle profiles of *atr.g* mutant and “Sebastian” were very similar. The same percentage of meristematic cells was arrested in the G2 phase in both genotypes after MH treatment, which suggests that in addition to the ATR-related pathway, another mechanism, probably ATM-related [1], may be (at least in part) involved in the DDR activation by maleic acid hydrazide.

Possibly the involvement of the DDR pathway in response to MH is linked with its inhibitory effects on S phase and accumulation of DNA breaks. Arabidopsis *atr* mutants are hypersensitive to replication-blocking agents such as hydroxyurea (HU), aphidicolin and UV-B light, and consequently show a greater than WT inhibition of root growth [19]. HU is an inhibitor of ribonucleotide reductase activity that is essential for production of dNTPs, aphidicolin is an inhibitor of DNA polymerases δ and ε and UV-B light causes pyrimidine dimers leading to physical replication blockage. The response of our *hvatr.g* mutant to MH differs from the response of Arabidopsis *atr* mutants to other replication-blocking agents. However, the response of Arabidopsis *atr* mutants to MH has not yet been reported. In our previous studies we showed that Al caused DNA damage in barley leading to cell cycle arrest [16]. However, in the *hvatr.g* mutant, despite the relatively high level of DNA damage (higher than in the “Sebastian”), the mitotic index in root meristem was not reduced and the progression of cell cycle was not affected by Al treatment [20]. Al is a mild genotoxic agent (at least in the tested doses, which correspond to physiological doses in acidic fields) and under Al treatment, impaired HvATR action led to maintenance of cell cycle progression despite DNA damage. MH used in this study is a highly genotoxic and clastogenic agent, and the *hvatr.g* mutant accumulated much higher levels of DNA damages after treatment with MH than with Al, which is consistent with Arabidopsis *atr* mutants being hypersensitive to other DNA damage agents that are highly toxic. A high level of MH-induced DNA damages led to a decrease in mitotic activity in the mutant, but still this reduction was much less relevant than in the case of WT cv. “Sebastian”. Additionally, MH changed the cell cycle profile of both genotypes—it caused the decrease of the cells in the G1 phase and the increase of cells in the G2/M phase. The treatment with Al affected the cell cycle profile only in “Sebastian”, while in the *hvatr.g* the frequency of different cell cycle phases remained similar as in control [20], which is consistent with Al acting as a mild genotoxin in Arabidopsis. The fact that cell cycle regulation seems to be affected by MH in the same way in WT and the ATR-deficient line suggests that the main signaling cascade leading to cell cycle arrest in the G2 phase in response to MH treatment relies on ATM. If it depends on ATR only, most cells would not be arrested in G2 but would escape and enter mitosis with unrepaired damage. However, the higher frequency of micronuclei in the mutant after MH treatment suggests that some cells escape this checkpoint and suggests potential co-participation of ATR and ATM in response to MH. The observed response of the *hvatr.g* mutant to Al and MH may indicate that the transduction of a signal of DNA damage does not function properly in the mutant, confirming the crucial role of ATR in the DDR pathway in barley.

Interestingly, the cell cycle profiles in root meristems of “Sebastian” and *hvatr.g* mutant grown under control conditions in this study were different from the profile described previously [20]. The frequency of G1 cells in this study was about 85% both in Sebastian and the *hvatr.g* mutant, whereas previously it was 24.4% and 21.5%, respectively. This may be due to the different culture conditions of the seedling growth, namely that the seedlings used in this study were grown in Petri dishes for 3 days, in the dark, whereas previously they were cultivated in hydroponics in Magnavaca solution at pH 4.0 for 7 days, in the light. The participation of the particular phases of the cell cycle is proved to be especially sensitive to changes in plant growth conditions [35].

## 4. Materials and Methods 

### 4.1. Plant Material 

The plant material used in the study was an *hvatr.g* mutant and spring barley (*Hordeum vulgare* L.) cultivar “Sebastian”, which is a parent variety of the *Hor*TILLUS (*Hordeum* TILLING University of Silesia) TILLING population developed at the Department of Genetics, University of Silesia in Katowice [22]. This population has been created after double treatment with sodium azide (NaN_3_) and N-methyl-N-nitrosourea (MNU) [36]. The *hvatr.g* mutant was identified as described by Szurman-Zubrzycka et al. [37] The plant material has been treated with aluminum (Al) or maleic acid hydrazide (MH) according to the procedures described below. 

### 4.2. MH Treatment 

Maleic acid hydrazide (4 mM MH; Sigma, CAS 123-3301) was used for mutagenic treatment. Before treatment, the seeds of *hvatr.g* mutant and cv. “Sebastian” were pre-soaked in distilled water for 8 h and then treated with MH for 3 h. After treatment, seeds were washed three times in distilled water and then germinated in Petri dishes at 21 °C in the dark for 72 h. The mutagenic treatment procedure was repeated twice. All treatment conditions used in the study were applied in the previous experiments in which cytogenetic effects of MH was estimated in barley [24].

### 4.3. Analysis of Mitotic Activity, the Frequency of Micronuclei and Damaged Nuclei 

Roots of barley seedlings, control and treated with MH, were used for cytogenetic studies. The mitotic activities, the frequency of nuclei with micronuclei and the frequency of damaged nuclei in the meristematic root cells of the *hvatr.g* mutant and “Sebastian” were analyzed. Roots were fixed in methanol: acetic acid (3:1 *v*/*v*) for 4 h at room temperature (RT). The treatment experiment was carried out in two biological repetitions, with three plants per repetition and three roots per each plant. Cytogenetic slides (each made from one root meristem) were prepared using the Feulgen’s squash technique. For each slide the above cytogenetic parameters were analyzed in 1000 root meristematic cells. Statistical analyses were performed using ANOVA (*p* < 0.05) followed by Tukey’s honestly significant difference test (Tukey HSD test, *p* < 0.05).

### 4.4. TUNEL Test 

The TUNEL (terminal deoxynucleotidyl transferase-mediated dUTP nick-end labeling) reaction was applied for the analysis of MH-induced DNA fragmentation. Control and MH-treated roots of the *hvatr.g* mutant and “Sebastian” were fixed in freshly prepared 4% paraformaldehyde (Fluka) in PBS (phosphate-buffered saline) for 1 h, at RT, and then washed 3 × 5 min in PBS. The nuclei preparations were made by squashing the meristematic tissue in the PBS buffer. After freezing at −70 °C, the slides were stored at 4 °C for several days. Prior to the TUNEL reaction, the slides were air-dried, permeabilized by incubating in 0.1% Triton X-100 (Sigma) in 0.1% sodium citrate at 4 °C for 2 min and were rinsed in PBS. For the positive control, a slide was treated with a DNAse solution (1U) for 30 min at 37 °C in a humid chamber. DNA fragment labelling was carried out with the TUNEL reaction mixture (in situ Cell Death Detection Kit, Fluorescein, Roche) containing an enzyme solution (terminal transferase) and a label solution (FITC labeled nucleotides). Next, 50 µL of the TUNEL reaction mixture (enzyme solution: label solution, 1:9 *v*/*v*) was applied to the preparations and incubated in a humid chamber for 1 h at 37 °C in the dark. As a negative control of the TUNEL reaction, a reaction mixture without any enzyme was used. The preparations were rinsed 3× in PBS and stained with DAPI (2 µg/mL), air dried and mounted in a Vectashield medium (Vector Laboratories). The frequency of TUNEL-positive nuclei was analyzed. The treatment experiment was carried out in two biological repetitions, with three plants per repetition and two roots per plant. The frequency of FITC-labelled nuclei in the TUNEL test was established based on the analysis of 2000 cells on each slide (each made from one root meristem). Preparations were examined with a Zeiss Axio Imager.Z.2 wide-field fluorescence microscope equipped with an AxioCam Mrm monochromatic camera. Statistical analyses were performed using ANOVA (*p* < 0.05) followed by Tukey’s honestly significant difference test (Tukey HSD test, *p* < 0.05). 

### 4.5. Cell Cycle Analysis using Flow Cytometry

For each experimental combination, approximately 30–50 root meristems of the *hvatr.g* mutant and “Sebastian”, control and MH-treated were analyzed. After mechanical root tip fragmentation, the suspension of nuclei was filtered through a 30-um nylon mesh to remove any large debris and then stained with staining buffer (CyStain^®^ UV Precise P, 05-5002, Sysmex). Samples were incubated for 12 min and analyzed using a CyFlow Space Sysmex flow cytometer with a 365 nm UV LED diode as the light source. Two samples were analyzed for each experimental group and the flow rate was adjusted to 20–40 nuclei per second. The results are shown on histograms prepared using a linear scale. For cell cycle phase determination, the software FloMax with the Cell Cycle Analysis application was used.

## 5. Conclusions

In light of the results presented, it can be concluded that the ATR-dependent DDR pathway plays a role in response to maleic acid hydrazide and thus demonstrates that this ATR-dependent mechanism is shared in plants in relation to a wide range of genotoxic agents including Al. Additionally, the *hvatr.g* mutant, with the ATR-dependent activation of DDR response, may serve as a useful tool in general studies on DNA repair processes induced by different factors in crop species.

## References

[1] Hu Z., Cools T., De Veylder L. (2016). Mechanisms Used by Plants to Cope with DNA Damage. Annu. Rev. Plant Biol..

[2] Lanier C., Bernard F., Dumez S., Leclercq-Dransart J., Lemière S., Vandenbulcke F., Nesslany F., Platel A., Devred I., Hayet A. (2019). Combined toxic effects and DNA damage to two plant species exposed to binary metal mixtures (Cd/Pb). Ecotoxicol. Environ. Saf..

[3] Mahapatra K., Roy S. (2020). An insight into the mechanism of DNA damage response in plants—Role of SUPPRESSOR OF GAMMA RESPONSE 1: An overview. Mutat. Res. Fundam. Mol. Mech. Mutagen..

[4] Delhaize E., Ryan P.R. (1995). Aluminum Toxicity and Tolerance in Plants. Plant Physiol..

[5] Kochian L., Piñeros M., Hoekenga O. (2005). The Physiology, Genetics and Molecular Biology of Plant Aluminum Resistance and Toxicity. Plant Soil.

[6] Kochian L., Piñeros M., Liu J., Magalhaes J. (2015). Plant Adaptation to Acid Soils: The Molecular Basis for Crop Aluminum Resistance. Annu. Rev. Plant Biol..

[7] Zhang Y., Guo J., Chen M., Li L., Wang L., Huang C.-F. (2018). The Cell Cycle Checkpoint Regulator ATR Is Required for Internal Aluminum Toxicity Mediated Root Growth Inhibition in Arabidopsis. Front. Plant Sci..

[8] Chen P., Sjorgen C.A., Larsen P.B., Schnittger A. (2019). A multi-level response to DNA damage induced by aluminium. Plant J..

[9] Jaskowiak J., Kwasniewska J., Milewska-Hendel A., Kurczynska E.U., Szurman-Zubrzycka M., Szarejko I. (2019). Aluminum Alters the Histology and Pectin Cell Wall Composition of Barley Roots. Int. J. Mol. Sci..

[10] Riaz M., Yan L., Wu X., Hussain S., Aziz O., Jiang C. (2018). Mechanisms of organic acids and boron induced tolerance of aluminum toxicity: A review. Ecotoxicol. Environ. Saf..

[11] Eekhout T., Larsen P., De Veylder L. (2016). Modification of DNA Checkpoints to Confer Aluminum Tolerance. Trends Plant Sci..

[12] Rounds M., Larsen P. (2008). Aluminum-Dependent Root-Growth Inhibition in Arabidopsis Results from AtATR-Regulated Cell-Cycle Arrest. Curr. Biol..

[13] Culligan K.M., Robertson C.E., Foreman J., Doerner P., Britt A.B. (2006). ATR and ATM play both distinct and additive roles in response to ionizing radiation. Plant J..

[14] Nisa M.U., Huang Y., Benhamed M., Raynaud C. (2019). The Plant DNA Damage Response: Signaling Pathways Leading to Growth Inhibition and Putative Role in Response to Stress Conditions. Front. Plant Sci..

[15] Nezames C., Sjogren C., Barajas J., Larsen P. (2012). The Arabidopsis Cell Cycle Checkpoint Regulators TANMEI/ALT2 and ATR Mediate the Active Process of Aluminum-Dependent Root Growth Inhibition. Plant Cell.

[16] Jaskowiak J., Tkaczyk O., Slota M., Kwasniewska J., Szarejko I. (2018). Analysis of aluminium toxicity in Hordeum vulgare roots with an emphasis on DNA integrity and cell cycle. PLoS ONE.

[17] Sjogren C., Bolaris S., Larsen P. (2015). Aluminum-Dependent Terminal Differentiation of the Arabidopsis Root Tip Is Mediated through an ATR-, ALT2-, and SOG1-Regulated Transcriptional Response. Plant Cell.

[18] Sjogren C., Larsen P. (2017). SUV2, which encodes an ATR-related cell cycle checkpoint and putative plant ATRIP, is required for aluminium-dependent root growth inhibition in Arabidopsis. Plant Cell Environ..

[19] Culligan K., Tissier A., Britt A. (2004). ATR Regulates a G2-Phase Cell-Cycle Checkpoint in Arabidopsis thaliana. Plant Cell.

[20] Szurman-Zubrzycka M., Nawrot M., Jelonek J., Dziekanowski M., Kwasniewska J., Szarejko I. (2019). ATR, a DNA Damage Signaling Kinase, Is Involved in Aluminum Response in Barley. Front. Plant Sci..

[21] Staub E., Fiziev P., Rosenthal A., Hinzmann B. (2004). Insights into the evolution of the nucleolus by an analysis of its protein domain repertoire. BioEssays.

[22] Szurman-Zubrzycka M., Zbieszczyk J., Marzec M., Jelonek J., Chmielewska B., Kurowska M., Krok M., Daszkowska-Golec A., Guzy-Wróbelska J., Gruszka D. (2018). HorTILLUS—A Rich and Renewable Source of Induced Mutations for Forward/Reverse Genetics and Pre-breeding Programs in Barley (*Hordeum vulgare* L.). Front. Plant Sci..

[23] Rank J., Lopez L.C., Nielsen M., Moretton J. (2002). Genotoxicity of maleic hydrazide, acridine and DEHP in Allium cepa root cells performed by two different laboratories. Hereditas.

[24] Juchimiuk J., Hering B., Maluszynska J. (2007). Multicolour FISH in an analysis of chromosome aberrations induced by *N*-nitroso-*N*-methylurea and maleic hydrazide in barley cells. J. Appl. Genet..

[25] Kwasniewska J., Grabowska M., Kwasniewski M., Kolano B. (2012). Comet-FISH using rDNA probes in analysis of mutagen-induced DNA damage in plant cells. Environ. Mol. Mutagen..

[26] Kwasniewska J., Kwasniewski M. (2013). Comet-FISH for the evaluation of plant DNA damage after mutagenic treatments. J. Appl. Genet..

[27] Michaelis A., Rieger R. (1968). On the distribution between chromosomes of chemically induced chromatid aberrations: Studies with a new karyotype of Vicia faba. Mutat. Res. Fundam. Mol. Mech. Mutagen..

[28] Nicoloff H., Rieger R., Michaelis A. (1979). Deletion clustering in specific chromosome segments of *Hordeum vulgare* and *Vicia faba*. Biol. Zentralbl..

[29] Maluszynska J., Maluszynski M. (1983). The influence of MNUA and MH on the cell cycle and DNA contents in meristematic cells of barley. Acta Biol..

[30] Marcano L., Carruyo I., Del Campo A., Montiel X. (2004). Cytotoxicity and mode of action of maleic hydrazide in root tips of *Allium cepa* L.. Environ. Res..

[31] Jabee F., Ansari M.Y., Shahab D. (2008). Studies on the Effect of Maleic Hydrazide on Root Tip Cells and Pollen Fertility in *Trigonella foenum-graceum* L.. Turk. J. Bot..

[32] Kwasniewska J., Kus A., Swoboda M., Braszewska-Zalewska A. (2016). DNA replication after mutagenic treatment in Hordeum vulgare. Mutat. Res. Genet. Toxicol. Environ. Mutagen..

[33] Kim J., Ryu T., Lee S., Chung B. (2019). Ionizing radiation manifesting DNA damage response in plants: An overview of DNA damage signaling and repair mechanisms in plants. Plant Sci..

[34] Kwasniewska J., Zubrzycka K., Kus A. (2018). Impact of Mutagens on DNA Replication in Barley Chromosomes. Int. J. Mol. Sci..

[35] Seaton D.D., Krishnan J. (2016). Model-Based Analysis of Cell Cycle Responses to Dynamically Changing Environments. PLoS Comput. Biol..

[36] Szarejko I., Szurman-Zubrzycka M., Nawrot M., Marzec M., Gruszka D., Kurowska M., Chmielewska B., Zbieszczyk J., Jelonek J., Maluszynski M. (2017). Creation of a TILLING in Barley After Chemical Mutagenesis with Sodium Azide and MNU. Biotechnologies for Plant Mutation Breeding.

[37] Szurman-Zubrzycka M., Chmielewska B., Gajewska P., Szarejko I. (2017). Mutation Detection by Analysis of DNA Heteroduplexes in TILLING Populations of Diploid Species. Biotechnologies for Plant Mutation Breeding.

